# Global proteome profiling of human livers upon ischemia/reperfusion treatment

**DOI:** 10.1186/s12014-020-09310-w

**Published:** 2021-01-06

**Authors:** Haijian Cai, Shunli Qi, Qi Yan, Jun Ling, Jian Du, Lijian Chen

**Affiliations:** 1 Department of Biochemistry and Molecular Biology, School of Basic Medical Sciences, Anhui Medical University, Hefei, Anhui 230032 People’s Republic of China; 2 The Key Laboratory of Pathogen Biology of Anhui Province, Anhui Medical University, Hefei, 230032 China; 3 Department of Anesthesiology, the First Affiliated Hospital of Anhui Medical University, Hefei, Anhu 230032 People’s Republic of China

**Keywords:** Hepatic ischemia/reperfusion, Human, Proteomics, TMT

## Abstract

Hepatic ischemia/reperfusion (I/R) injury represents a major risk factor for liver transplantation and is related to graft dysfunction and acute/chronic rejection. However, a significant part of these processes remain poorly characterized. To reveal differences in the proteome during liver I/R injury, we collected human liver biopsy samples during hepatectomy before and after the Pringle maneuver and conducted a TMT-based proteomic analysis through quantitative high-throughput mass spectrometry. We used a fold-change threshold of 1.3 and a *t*-test *p*-value < 0.05 as the criteria to identify 5,257 total quantifiable proteins. The levels of 142 proteins were increased, while the levels of 103 proteins were decreased in response to hepatic I/R treatment. Bioinformatic analysis further revealed that these differentially expressed proteins are mainly involved in multiple biological functions and enzyme-regulated metabolic pathways. Most proteins whose expression was changed are related to the defense, immune and inflammatory responses as well as lipid and steroid metabolic processes. Based on this finding, we developed a panel for targeted proteomic analysis and used the parallel reaction monitoring (PRM) method, qPCR and western blotting experiments to validate alterations in the expression of some of the identified proteins. The upregulated proteins were found to be involved in immunity and inflammatory responses, and downregulated proteins were enriched in metabolic pathways. This study therefore may provide a research direction for the design of new therapeutic strategies for hepatic ischemia/reperfusion injury.

## Introduction

Severe intraoperative bleeding represents a major risk during hepatectomy. To avoid massive blood loss, continuous or intermittent vascular clamping of the hepatic artery and portal vein, the so-called Pringle maneuver, is an efficient method to reduce hemorrhage. However, the Pringle maneuver can lead directly to reperfusion in the liver. Liver ischemia/reperfusion (I/R) injury is a source of morbidity and mortality of liver surgeries, such as hepatectomy and transplantation, hemorrhagic shock and tissue resection [1, 2]. Ischemia/reperfusion injury (IRI) is tissue damage caused when blood supply returns to tissue (reperfusion) after a period of ischemia or lack of oxygen (anoxia or hypoxia). Two distinct stages of liver IRI with unique mechanisms of hepatic damage have been identified. Ischemic injury, a process of localized cellular metabolic disturbance, results from glycogen consumption, a lack of the oxygen supply and ATP depletion and lead to initial parenchymal cell death. During an ischemic period, hepatocytes switch their cellular metabolism from aerobic to anaerobic pathways, which causes various hepatocellular dysfunctions. Reperfusion injury, which follows ischemic injury, results from not only metabolic disturbances but also a profound inflammatory immune response that involves both direct and indirect cytotoxic mechanisms [3]. The absence of oxygen and nutrients in the blood during the ischemic period creates a condition in which the restoration of circulation results in inflammation and oxidative damage through the induction of oxidative stress rather than the restoration of normal function. Several factors and mechanisms implicated in the hepatic IRI process are anaerobic metabolism, mitochondria damage, oxidative stress and ROS, intracellular calcium overload, liver Kupffer cells and neutrophils, nitric oxide, cytokines and chemokines [3–7]. Indeed, liver IRI remains a major problem in clinical transplantation, causing more than 10% of early transplant failures and leading to a higher incidence of both acute and chronic rejection [8, 9]. Despite the obvious clinical importance of liver I/R, the mechanisms that account for I/R injury are only partially understood and remain one of the most understudied areas in clinical and experimental transplantation [3].

To understand the molecular bases of liver I/R-induced injury and develop novel approaches for injury control, it is important to identify genes/proteins whose expression/function is directly modified by the damage itself. However, due to limited knowledge of the functional molecules involved in I/R injury and the complexity of the regulatory mechanisms of the liver response, the identification of molecular targets for therapeutic intervention is very difficult. Nevertheless, rapid progress in both protein separation and identification techniques has made mass spectrometry a powerful tool with which to characterize proteins in a more global way by the simultaneous evaluation of hundreds of protein species, allowing the study of biological responses in complex systems [10–12]. In the present study, we performed a quantitative proteomics study of hepatic hemangioma patients who underwent hepatectomy before and after the Pringle maneuver. Here, we found that 142 proteins were significantly upregulated and that 103 proteins were downregulated upon I/R treatment. These findings may assist in designing new therapeutic strategies for hepatectomy, including organ preservation for liver transplantation.

## Methods

### Sample collection and preparation

Six patients with hepatic hemangioma underwent hepatectomy receiving Pringle maneuver which caused hepatic ischemia reperfusion injury (mean age 50.3, range 36–65). The median ischemia times were 17 min (mean time 17 min, range 9–22 min), and the reperfusion times were all 10 min. The liver samples (0.8–1.0 cm^3^) were obtained away from the resection site of the liver prior to Pringle maneuver (control group) and 10 min after restoration of blood supply (ischemia/reperfusion group) respectively during the surgery, and snapped frozen on liquid nitrogen for 1–2 min, then moved quickly and stored at − 80 °C until analysis. An informed written consent was obtained from all patients. The patients were recruited at the First Affiliated Hospital of Anhui Medical University and approved by the institutional ethics committee (No: 20180145).

### Protein extraction

The sample was sonicated three times on ice using a high intensity ultrasonic processor (Scientz) in four volumes of lysis buffer (8 M urea, 300 mM Tris pH8, 4% CHAPS, 1MNaCl, 1% Protease Inhibitor Cocktail). The remaining debris was removed by centrifugation at 12,000 g at 4 °C for 10 min. Finally, the supernatant was collected and the protein concentration was determined with BCA kit according to the manufacturer’s instructions.

### Digestion and TMT labeling

For digestion, the protein solution was reduced with 5 mM dithiothreitol for 30 min at 56 °C and alkylated with 11 mM iodoacetamide for 15 min at room temperature in darkness. The protein sample was then diluted by adding 100 mM TEAB to urea concentration less than 2 M. Finally, trypsin was added at 1:50 trypsin-to-protein mass ratio for the first digestion overnight and 1:100 trypsin-to-protein mass ratio for a second 4 h-digestion. After trypsin digestion, peptide was desalted by Strata X C18 SPE column (Phenomenex) and vacuum-dried. Peptide was reconstituted in 0.5 M TEAB and processed according to the manufacturer’s protocol for TMT kit. Briefly, one unit of TMT reagent was thawed and reconstituted in acetonitrile. The peptide mixtures were then incubated for 2 h at room temperature and pooled, desalted and dried by vacuum centrifugation.

### HPLC fractionation

The protein digestion was then fractionated by high pH reverse-phase HPLC using Agilent 300 Extend C18 column (5 μm particles, 4.6 mm ID, 250 mm length). Briefly, peptides were first separated with a gradient of 2% to 60% acetonitrile in 10 mM ammonium bicarbonate pH 8 over 80 min into 80 fractions. Then, the peptides were combined into 18 fractions for the global proteome analysis as previously reported [13]. The tryptic peptides were fractionated into fractions by high pH reverse-phase HPLC using Agilent 300 Extend C18 column (5 μm particles, 4.6 mm ID, 250 mm length). Briefly, peptides were first separated with a gradient of 8% to 32% acetonitrile (pH 9.0) over 60 min into 60 fractions. Then, the peptides were combined into 18 fractions and dried by vacuum centrifuging.

### LC–MS/MS analysis

The peptides were dissolved in solvent A (0.1% FA in 2% ACN), directly loaded onto a home-made reversed-phase analytical column (15 cm length, 75 μm i.d.). The gradient was comprised of an increase from 6 to 23% solvent B (0.1% formic acid in 98% acetonitrile) over 26 min, 23% to 35% in 8 min and climbing to 80% in 3 min then holding at 80% for the last 3 min, all at a constant flow rate of 400 nL/min on an EASY-nLC 1000 UPLC system. The peptides were subjected to NSI source followed by tandem mass spectrometry (MS/MS) in Q Exactive™ Plus (Thermo) coupled online to the UPLC. The applied electrospray voltage was 2.0 kV. The m/z scan range was from 350 to 1,800 for full scan, and intact peptides were detected in the Orbitrap at a resolution of 70,000. Peptides were then selected for MS/MS using NCE setting as 28 and the fragments were detected in the Orbitrap at a resolution of 17,500. A data-dependent procedure was alternated between one MS scan followed by 20 MS/MS scans with 15.0 s dynamic exclusion. Automatic gain control (AGC) was set at 5E4. Fixed first mass was set as 100 m/z. Secondary mass spectral data was retrieved by using Maxquant (v1.5.2.8). Search parameter settings: the database was SwissProt Human (20,317 sequences), the anti-library was added to calculate the false positive rate (FDR) caused by random matching, and a common pollution library was added to the database to eliminate the contaminated protein in the identification results. The effect was set to Trypsin/P; the number of missed sites was set to 2; the minimum length of the peptide was set to 7 amino acid residues; the maximum number of peptides was set to 5; the primary precursor of the First search and Main search mass error tolerance was set to 20 ppm and 5 ppm respectively, and the mass error tolerance of the secondary fragment ions was 0.02 Da. The cysteine alkylation was set to a fixed modification, the variable modification was the oxidation of methionine and the N-terminal acetylation of the protein. The quantitative method was set to TMT-6 plex, and the FDR for protein identification and PSM identification was set to 1%.

### Statistical analysis

Data were expressed as mean ± standard deviation, and the differences between groups were analyzed using Student’s *t* test, *p* values < 0.05 or 0.01 were considered to represent statistical differences or statistically significant differences. In order to obtain bioinformatics, the UniProt-GOA database (http://www.ebi.ac.uk/GOA/) and the PANTHER (www.pantherdb.org) database were used.

### PRM

The peptide samples were prepared according to the whole cell proteome analysis methodology described above. The proteins selected for PRM validation were based on the results of whole cell proteome. In addition, to determine the target peptides of each protein selected for PRM and their retention times, the peptide sample was initially run in a DDA mode using Q Exactive™ Plus (Thermo Fisher Scientific, Waltham, MA, USA), which was coupled to the UPLC online with the identical gradient to subsequent PRM analysis. Based on the results of this pre-experiment, peptides were selected and input as entries into the inclusion list, which would be detected in the PRM assay. In the subsequent PRM analysis, the peptide mixture was loaded onto an PicoFrit capillary column (75 µm × 15 cm, New Objective, Woburn, MA, USA) packed with ReproSil-Pur Basic C18 reverse phase resin (1.9 µm, 100A°, Dr. Maisch GmbH, Ammerbuch, Germany) and separated in an EASY-nLC 1000 UPLC system (Thermo Fisher Scientific, Waltham, MA, USA), with a gradient of 6% to 23% solvent B (0.1% formic acid in 98% ACN) over 38 min, followed by 23–35% solvent B over 14 min and increasing to 80% solvent B over 4 min, with a flow rate of 400 nL/min. The eluate was examined via mass spectrometry using Q Exactive™ Plus (Thermo Fisher Scientific, Waltham, MA, USA), which was coupled to the UPLC online. After a full-scan event, the MS/MS scans in PRM mode were triggered by inclusion list. A full mass spectrum was detected in the Orbitrap at a resolution of 70,000 (AGC target was set as 3E^6^; the maximum injection time was 50 ms; and the m/z range was 350–1200), followed by 20 MS/MS scans on the Orbitrap at a resolution of 17,500 (AGC target was 1E^5^, and the maximum injection time was 100 ms) in a data independent procedure. Mass window for precursor ion selection was 1.6 m/z. The isolation window for MS/MS was set at 2.0 m/z. The NCE was 27% with HCD. Three biological replicates were performed. PRM data were analyzed using Skyline (v.3.6) software. The following parameters were set for this analysis: (1) enzyme, trypsin [KR/P]; (2) max missed cleavages, 0; (3) peptide length, 7–25; (4) static modification, Cys carbamidomethyl; (5) variable modification, Met oxidation; and (6) max variable modifications, 3. The following transition settings were used: (1) precursor charges, 2 and 3; (2) ion charges, 1 and 2; (3) ion types, b, y and p; (4) product ions, from ion 3 to the last ion; and (5) ion match tolerance, 0.02 Da. The peptide and protein information has been provided as Additional file 4: Table S4.

### Quantitative real-time RT-PCR

Total RNA for real-time quantitative RT-PCR was isolated from tissues using RNAiso Reagent (TaKaRa, Tokyo, Japan) and reverse transcripted into the single strand cDNA. Primers were designed using the Primer Express software version 1.5 (Applied Biosystems, Foster City, CA, USA) (Additional file 2: Table S2). Quantification of mRNA was performed using the Thermo Q50 (Thermo Fisher Scientific) with PrimeScript™ RT-PCR Kit (TaKaRa, Tokyo, Japan).

### Western blotting

Liver samples were homogenized in lysis buffer (10 mM HEPES, pH 7.9, 150 mM NaCl, 1 mM EDTA, 0.6% NP-40, 0.5 mM PMSF). Samples were then sonicated and incubated for 30 min on ice. Cellular debris was removed by centrifugation at 10,000 rpm. Protein concentrations of each sample were determined. Samples containing equal amounts of protein in equal volumes of sample buffer were separated in a denaturing 10% polyacrylimide gel and transferred to a 0.1 μm pore nitrocellulose membrane. Nonspecific binding sites were blocked with tris-buffered saline (TBS; 40 mM Tris, pH 7.6, 300 mM NaCl) containing 5% non-fat dry milk for 1 h at room temperature. Membranes were then incubated with antibodies to AXL (1:1000, CST, C89E7) or ECM1 (1:1000, Proteintech, 11,521–1-AP) in TBS with 0.1% Tween 20 (TBST). Membranes were washed and incubated with secondary antibodies conjugated to horseradish peroxidase. Immunoreactive proteins were detected by enhanced chemiluminescence.

## Results

### Stepwise workflow based on TMT-based LC–MS/MS to select and evaluate the protein signatures of the hepatic I/R and control groups

**Fig. 1:**
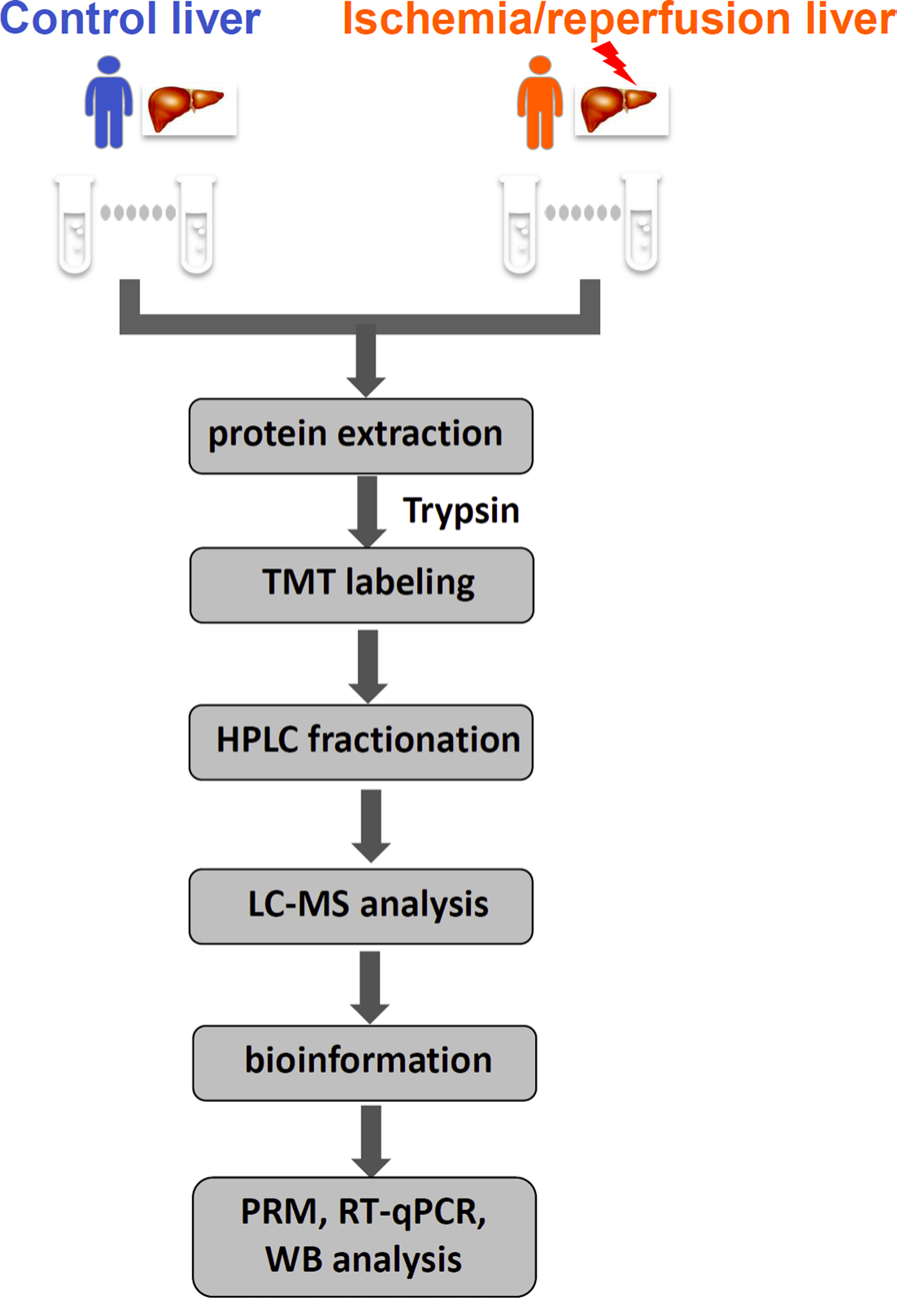
Flowchart detailing the experiments including the sample preparation, data acquisition, and data analysis performed in this study. The protein peptides digested by liver biopsies were collected from six patients with hepatic hemangioma underwent hepatectomy prior to Pringle maneuver (Control group) and 10 min after restoration of blood supply (Ischemia/reperfusion group) respectively, then these samples were subjected to TMT labeling, HPLC fractionation and biological analysis after LC–MS/MS, and verified by PRM, qPCR and western blotting

**Fig. 2:**
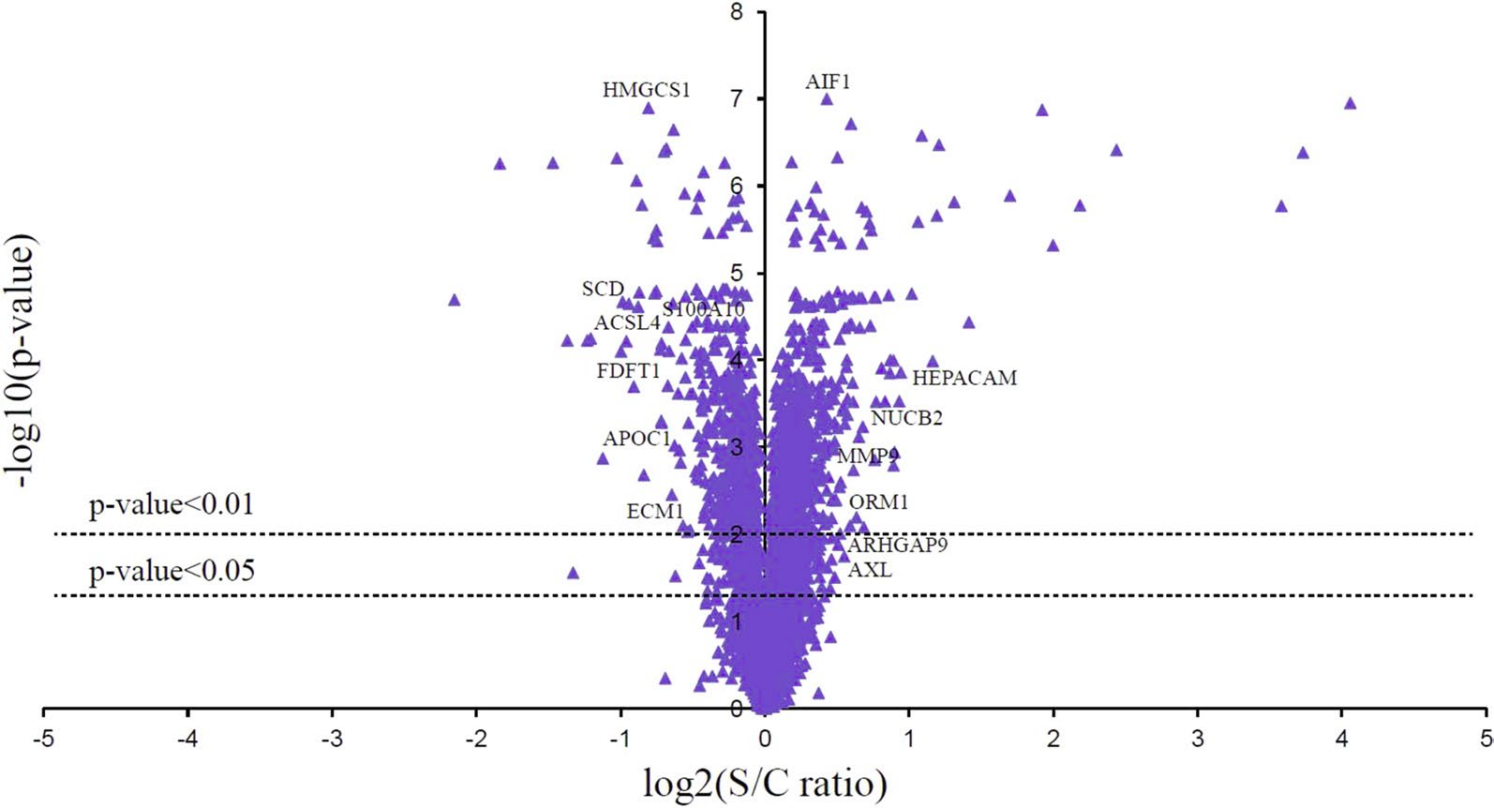
Volcano plot of proteins with the most significantly different abundance levels between ischemia/reperfusion and control livers. Negative log_10_ *p*-values are plotted logarithmically against the log_2_ protein ratios. Increased protein levels are presented on the positive X-axis, and decreased levels are presented on the negative X-axis. The differentially expressed proteins of *p* value < 0.05 and *p* value < 0.01 were detailed respectively

For the purpose of this study, liver biopsy samples were collected from hepatic hemangioma patients who underwent hepatectomy prior to the Pringle maneuver (Control group) and 10 min after the restoration of blood supply (I/R group). Then, TMT labeling, high-performance liquid chromatography, and mass spectrometry-based quantitative proteomics were carried out. The general experimental strategy is illustrated in Fig. 1. A total of 5257 proteins were quantitatively identified in samples from the I/R and control groups. Compared with the control group, 245 proteins displayed significant changes in their expression levels in the I/R group. Of these total proteins, 142 proteins were upregulated (the right part of the axis), and 103 proteins were downregulated (the left part of the axis). Differentially expressed proteins with *p*-values < 0.05 and those with *p*-values < 0.01 are detailed in Fig. 2. The list of significantly regulated proteins, together with their log_2_ changes, corresponding *p*-values, and relevant biological processes, are also shown in Table 1 (more details are provided in Additional file 1: Table S1).

**Table 1:** List of significantly regulated proteins in ischemia/reperfusion group versus control group using LC–MS/MS approach

No.	Protein accession	Protein name	Gene name	Log_2_ of av. ratio	*p*-value	Biological process
1	P00973	2′-5′-Oligoadenylate synthetase 1	OAS1	− 0.225↓	0.00002	Activate of latent RNase L and induce viral RNA degradation
2	Q2TB90	Hexokinase domain containing 1	HKDC1	− 0.361↓	0.00000	A member of the hexokinase involved in glucose metabolism
3	Q96AM1	MAS related GPR family member F	MRGPRF	− 0.426↓	0.00006	Regulate nociceptor function and/or development
4	Q9UBS3	DnaJ (Hsp40) homolog	DNAJB9	− 0.433↓	0.00006	Induced by ER stress and protect stressed cells from apoptosis
5	O00767	Stearoyl-CoA desaturase	SCD	− 0.491↓	0.00002	An enzyme involved in fatty acid biosynthesis
6	Q9UNA1	Rho GTPase activating protein 26	ARHGAP26	− 0.54↓	0.00000	A GTPase activating protein that binds to focal adhesion kinase
7	P20591	Interferon-inducible protein P78	MX1	− 0.544↓	0.00002	A GTP-metabolizing protein involved in the cellular antiviral response
8	Q01581	HMG-CoA synthase	HMGCS1	− 0.572↓	0.00000	Regulation of cholesterol biosynthesis
9	P05161	ISG15 ubiquitin-like modifier	ISG15	− 0.588↓	0.00002	A ubiquitin-like protein upon activation by IFN-α and IFN-β
10	P37268	Squalene synthase	FDFT1	− 0.609↓	0.00006	Catalyze the reaction to form squalene in cholesterol biosynthesis
11	Q14397	Glucokinase regulator	GCKR	− 0.609↓	0.00052	A regulatory protein that inhibits glucokinase in liver
12	Q8N126	Cell adhesion molecule 3	CADM3	− 0.631↓	0.00008	A calcium-independent cell–cell adhesion protein
13	Q9Y6K5	Interferon-induced protein 56	IFIT1	− 0.643↓	0.00002	Inhibit expression of viral messenger RNAs
14	Q9UK22	F-box protein 2	FBXO2	− 0.664↓	0.00108	Function in phosphorylation-dependent ubiquitination
15	Q9TQE0	MHC class II HLA-DR beta 1 chain	HLA-DRB1	− 0.683↓	0.00016	Present peptides derived from extracellular proteins
16	O60488	Long-Chain Acyl -CoA synthetase 4	ACSL4	− 0.684↓	0.00006	Play a key role in lipid biosynthesis and fatty acid degradation
17	O75604	Ubiquitin specific peptidase 2	USP2	− 0.693↓	0.00052	A ubiquitin-specific protease required for TNF-α-induced NF-κB
18	Q16610	Extracellular matrix protein 1	ECM1	− 0.698↓	0.00896	Involved in angiogenesis and tumor biology
19	Q9NR19	Acetyl-coenzyme a synthetase 2	ACSS2	− 0.717↓	0.00008	Activation of acetate for use in lipid synthesis and energy generation
20	Q15800	Methylsterol monooxygenase 1	MSMO1	− 0.72↓	0.00000	Localized to the ER membrane function in cholesterol biosynthesis
21	P11509	Cytochrome P450 family 2	CYP2A6	− 0.722↓	0.00004	Involved in of cholesterol, steroids and other lipids
22	Q8TF05	Phosphatase 4 regulatory subunit 1	PPP4R1	− 0.729↓	0.02137	Serine/threonine protein phosphatase 4 (PP4) to regulate HDAC3
23	P14555	Phospholipase A2 group IIA	PLA2G2A	− 0.74↓	0.00038	Participate in phospholipid metabolism in biomembranes
24	P07355	Annexin A2	ANXA2	− 0.746↓	0.00638	Members of this calcium-dependent phospholipid-binding protein family
25	O94760	Dimethylargininase-1	DDAH1	− 0.751↓	0.00504	Plays a role in nitric oxide generation
26	Q9NYL2	MAPKKK20	MAP3K20	− 0.755↓	0.00034	A member of MAPKKK family and play a role in cell cycle checkpoint
27	P60903	S100 calcium binding protein A10	S100A10	− 0.758↓	0.00004	Involved in the regulation of cell cycle progression and differentiation
28	P02654	Apolipoprotein C1	APOC1	− 0.762↓	0.00068	Play a central role in HDL and VLDL metabolism
29	Q15582	Transforming growth factor β	TGFBI	− 0.769↓	0.00258	Modulate cell adhesion and serves as a ligand for several integrins
30	Q9H6E4	Coiled-coil domain containing 134	CCDC134	− 0.769↓	0.03265	Inhibit ERK and JNK signal to suppress cell migration and invasion
31	P02750	Leucine rich alpha-2-glycoprotein 1	LRG1	1.301↑	0.00032	Protein–protein interaction, and cell adhesion and development
32	P09917	Arachidonate 5-lipoxygenase	ALOX5	1.302↑	0.03072	Synthesis of leukotrienes to mediate inflammatory and allergy
33	P14780	Matrix metallopeptidase 9	MMP9	1.304↑	0.00132	Breakdown of extracellular matrix in tissue remodeling
34	P05164	Myeloperoxidase	MPO	1.306↑	0.00000	Host defense system of polymorphonuclear leukocytes
35	P09601	Heme oxygenase 1	HMOX1	1.309↑	0.00046	Cytoprotective effects against apoptosis
36	P30530	AXL receptor tyrosine kinase	AXL	1.317↑	0.02248	Tyrosine kinase receptor involved in growth, migration, aggregation
37	Q9BYE9	Cadherin related family member 2	CDHR2	1.322↑	0.00610	Calcium-dependent cell–cell adhesion molecules
38	Q9BRR9	Rho GTPase activating protein 9	ARHGAP9	1.325↑	0.01110	A member of the Rho-GAP family of GTPase activating proteins
39	P55290	Cadherin 13	CDH13	1.332↑	0.00762	Protects cells from apoptosis due to oxidative stress
40	P02776	Platelet factor 4	PF4	1.346↑	0.00314	A member of the CXC chemokine family
41	P55008	Allograft inflammatory factor 1	AIF1	1.347↑	0.00000	Play a role in macrophage activation and function
42	A0A075B6K5	Immunoglobulin lambda variable	IGLV3-9	1.357↑	0.00054	Participates in the antigen recognition
43	Q15165	Paraoxonase 2	PON2	1.375↑	0.00018	A cellular antioxidant, protecting cells from oxidative stress
44	Q9ULU4	Zinc finger MYND-type containing 8	ZMYND8	1.379↑	0.00650	Down-regulation of diverse metastasis-associated genes
45	Q8TD30	Glutamic–alanine transaminase	GPT2	1.381↑	0.01102	Catalyzes the reversible transamination between alanine and glutamate
46	P02763	Orosomucoid 1	ORM1	1.384↑	0.00396	An acute-phase reactant
47	Q9H799	Ciliogenesis and planar polarity effector 1	C5orf42	1.399↑	0.00052	Establishment of cell polarity required for directional cell migration
48	P09210	Glutathione S-transferase alpha 2	GSTA2	1.422↑	0.01306	Glutathione S-transferase
49	P05413	Fatty acid binding protein 3	FABP3	1.44↑	0.00254	Participate in intracellular metabolism of long-chain fatty acids
50	P80303	Nucleobindin 2	NUCB2	1.458↑	0.00034	Have a role in calcium homeostasis
51	Q96JA1	Leucine-rich repeat protein LRIG1	LRIG1	1.465↑	0.00002	A feedback negative regulator of signaling by receptor tyrosine kinases
52	Q5R3K3	Calcium homeostasis modulator 6	CALHM6	1.608↑	0.00832	Calcium homeostasis
53	O00479	High mobility group nucleosomal binding domain 4	HMGN4	1.629↑	0.00000	Reduce the compactness of the chromatin fiber in nucleosomes
54	Q14CZ8	Hepatocyte cell adhesion molecule	HEPACAM	1.923↑	0.00014	Involved in cell motility and cell–matrix interactions
55	P30711	Glutathione S-transferase Theta 1	GSTT1	2.087↑	0.00000	A member of glutathione s-transferase
56	O94885	SAM and SH3 domain containing 1	SASH1	2.283↑	0.00000	A scaffold protein involved in the tlr4 signaling pathway
57	Q86VW2	GTP-rho-binding protein 2	RHPN2	3.246↑	0.00000	A member of rho-gtpase binding proteins
58	P02741	C-reactive protein	CRP	5.412↑	0.00000	Acute phase response to tissue injury, infection, or inflammatory stimuli
59	Q03013	Glutathione S-transferase Mu 4	GSTM4	11.968↑	0.00000	A member of glutathione S-transferase
60	P09488	Glutathione S-transferase Mu 1	GSTM1	13.258↑	0.00000	A member of glutathione S-transferase

### Functional classification of differentially expressed proteins upon hepatic I/R treatment

**Fig. 3:**
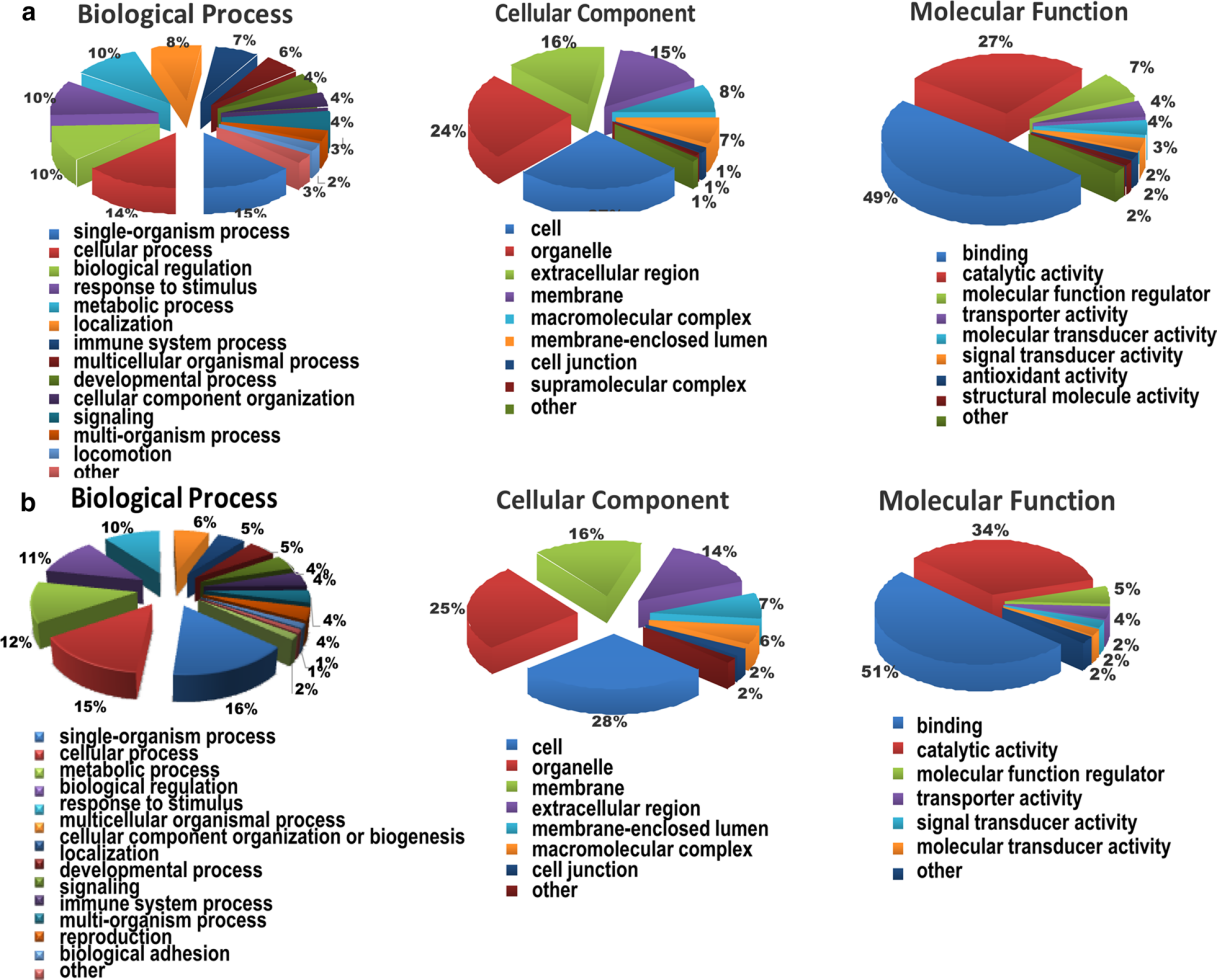
Functional classification of differentially expressed proteins upon hepatic I/R treatmemt. **a** Gene Ontology analysis of biological processes, molecular functions and cellular components of up-regulated proteins upon hepatic I/R using PANTHER tool. **b** Gene Ontology analysis of biological processes, molecular functions, and cellular components of down-regulated proteins upon hepatic I/R using PANTHER tool

To elucidate functional differences between the upregulated and downregulated proteins, the quantified proteins were analyzed by three types of enrichment-based clustering analyses: Gene ontology (GO) enrichment-based clustering analysis, protein domain enrichment-based clustering analysis and KEGG pathway enrichment-based clustering analysis. Gene ontology analysis of biological processes, cellular compartments and molecular functions was used to analyze the biological functions of the differentially expressed proteins. According to their molecular functions, these proteins whose expression was altered are strongly associated with binding and catalytic activity. In addition, a majority of the differentially expressed proteins were found to be located in cells and organelles (Fig. 3 and Additional file 3: Table S3). According to biological process analysis, upregulated proteins were mainly associated with single-organism and cellular processes and responses to stimuli (Fig. 3A). Downregulated proteins were mostly associated with single-organism, cellular and metabolic biological processes (Fig. 3B). By using protein domain enrichment-based clustering analysis, the results of which were consistent with those of molecular function and biological process analyses, we observed that upregulated proteins were mostly associated with immunoglobulin-like and Glutathione-S-transferase domains. Furthermore, the downregulated proteins were mainly associated with synthetase domains (Additional file 5: Figure S1). These results suggested that these differentially expressed proteins are mainly involved in biological processes and molecular functions, especially those in response to stimuli; cellular components; and metabolic processes.

### Enrichment analysis of differentially expressed proteins upon hepatic I/ R treatment

**Fig. 4:**
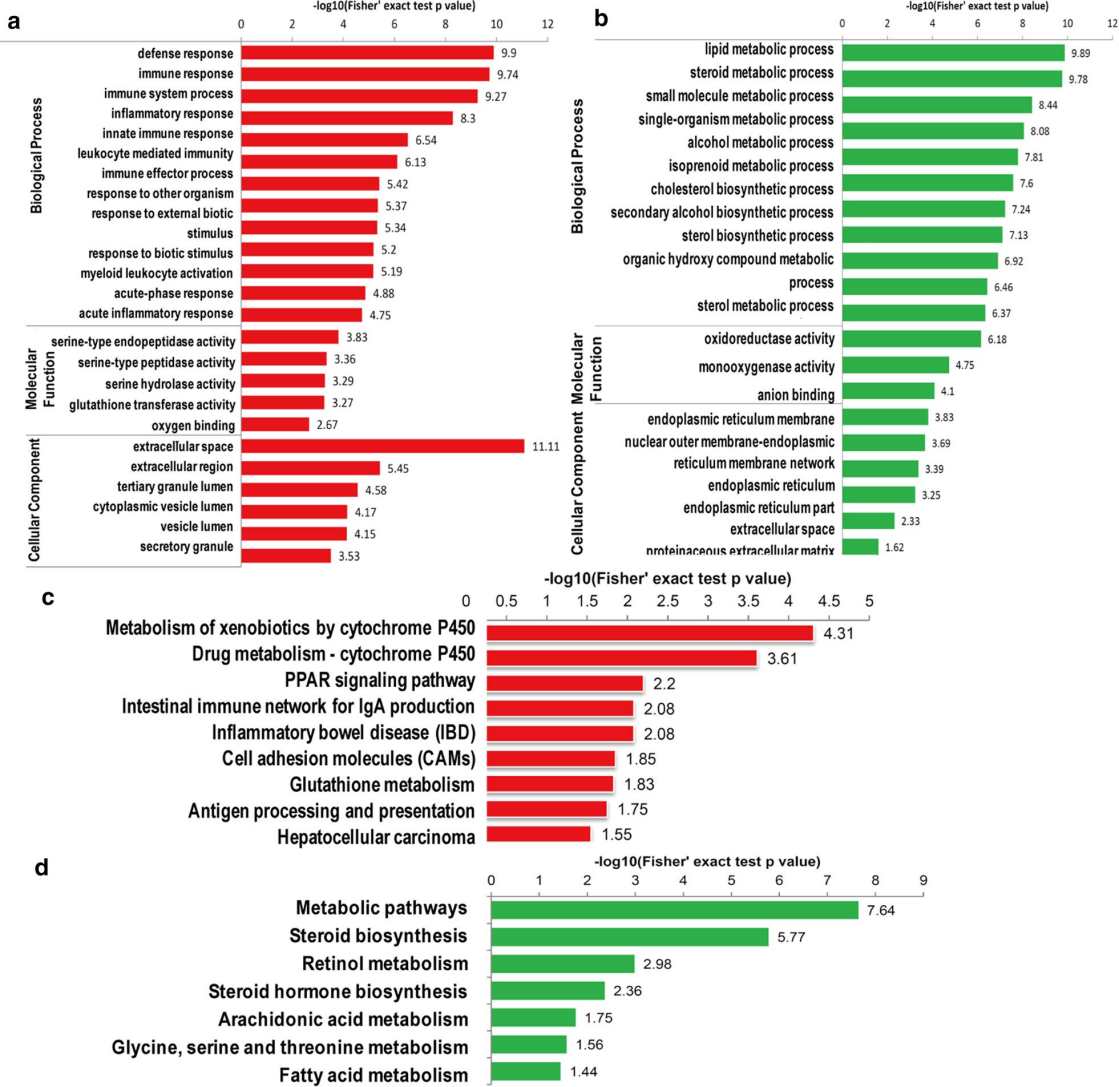
Enrichment analysis of differentially expressed proteins upon hepatic I/R treatment. **a** Functional enrichment analysis of up-regulated proteins upon hepatic I/R treatment. **b** Functional enrichment analysis of down-regulated proteins upon hepatic I/R treatment. **c** Functional enrichment analysis based on KEGG analysis of up-regulated proteins upon hepatic I/R treatment. **d** Functional enrichment analysis based on KEGG analysis of down-regulated proteins upon hepatic I/R treatment

According to gene ontology enrichment analysis, upregulated differentially expressed proteins were associated with the defense, immune and inflammatory response pathways (Fig. 4a). In terms of their molecular functions, these proteins were closely related to the binding of serine peptidases, hydrolases and glutathione transferase and oxygen binding. In addition, most of the differentially expressed proteins were distributed throughout the cell and closely related to secretory proteins. Downregulated proteins were mostly associated with metabolic processes, and their molecular functions were mainly related to oxidoreductase function (Fig. 4b). KEGG-based functional enrichment analysis was also performed and showed that the upregulated proteins were mainly related to the immune network and inflammation (Fig. 4Cc). Proteins that were downregulated after I/R were enriched in many important processes, including metabolic pathways, steroid biosynthesis, retinol metabolism, steroid hormone biosynthesis, arachidonic acid and fatty acid metabolism (Fig. 4d). For more details on the functions of the differentially expressed proteins after ischemia/reperfusion, cluster analysis based on GO and KEGG enrichment was performed (Additional file 6: Figure S2). We used a significant fold-change ratio of 1.3 and performed the above clustering analyses by dividing all proteins with significantly differential expression into four quantiles (Q1-Q4) according to their L/H ratios as follows: Q1 (≤ 0.77), Q2 (0.77 < ratio ≤ 0.83), Q3 (1.2 < ratio ≤ 1.3) and Q4 (> 1.3) to determine the biological functions of proteins with a large change in expression (1.3 or 0.77) or a relatively small change in expression (1.2–1.3 or 0.77–0.83) upon I/R treatment. The presence of functional pathway blocks and the functional descriptions of different sets of differentially expressed proteins indicated the degree of enrichment. Red indicates strong enrichment, and blue indicates weak enrichment. Biological process clustering analysis showed that the upregulated proteins were closely related to secretion, immunity, the response to stimuli and cytokines. In contrast, the downregulated proteins were mostly associated with metabolism (Additional file 6: Figure S2a). As shown by molecular functional clustering analysis, the altered proteins were closely related to receptor activity, binding, and transferase activity (Additional file 6: Fig. S2b). By cellular component clustering analysis, the upregulated proteins were found to be localized to the nuclear membrane and plasma membrane fractions, while the downregulated proteins were mainly localized to the endoplasmic reticulum and nuclear membrane (Additional file 6: Figure S2c). KEGG pathway clustering analysis showed that the upregulated proteins were involved in immune and inflammatory responses, such as the PPAR signaling pathway (Additional file 7: Figure S3). Downregulated proteins were mostly involved in various metabolic and biosynthetic pathways (Additional file 6: Figure S2d). Taken together, these data demonstrated that the upregulated proteins were involved in immunity and inflammatory responses and that the downregulated proteins were involved in metabolic pathways.

### Validation of differentially expressed proteins upon hepatic I/R using PRM, WB, and qPCR

**Fig. 5:**
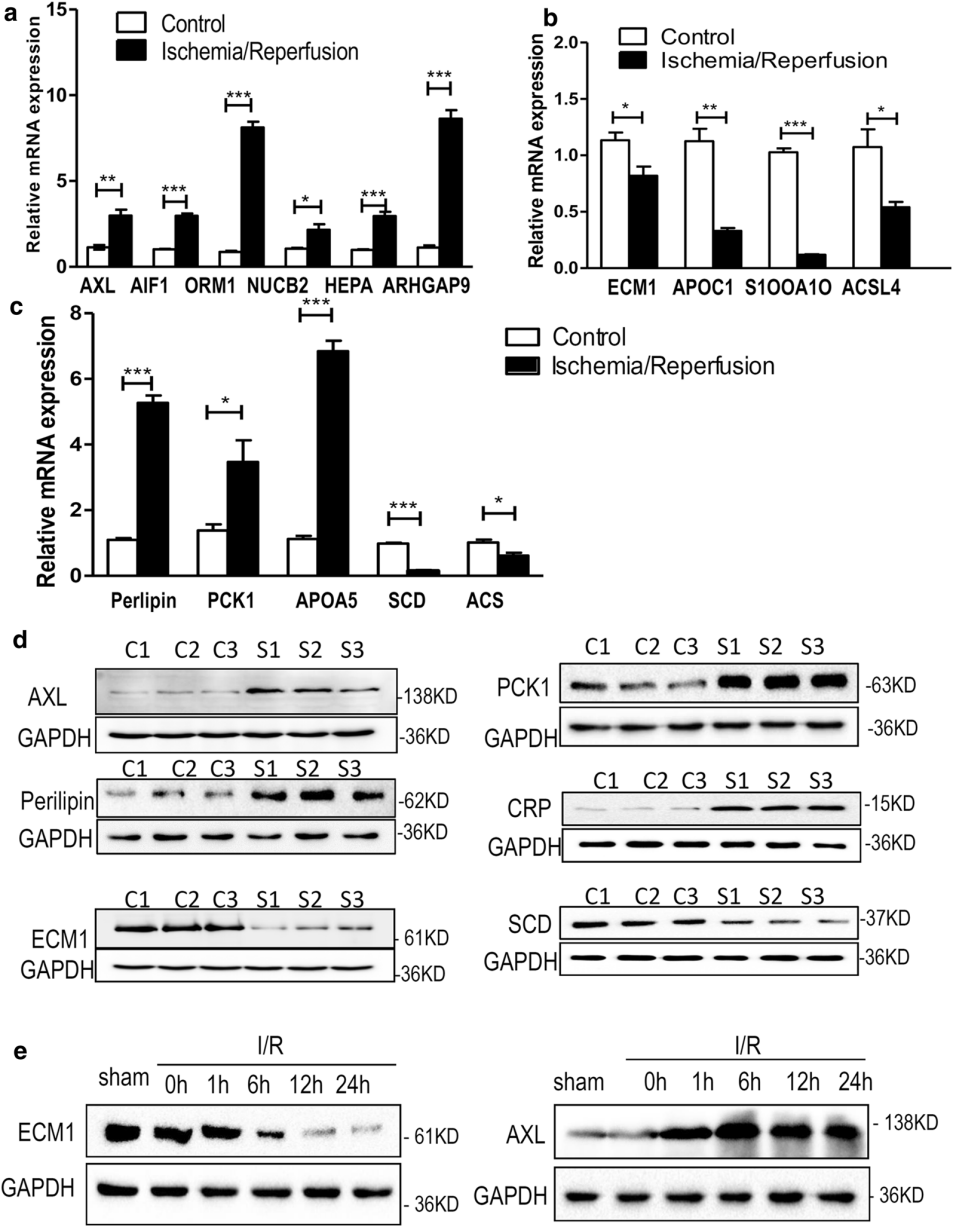
Validation of ischemic groups and control groups using western blotting and qPCR. **a** The mRNA expressions of up-regulated proteins, such as, AXL, AIF1, ORM1, NUCB2, HEPA, and ARHGAP9 of control and ischemia/reperfusion livers were verified using quantitative real-time analysis. The error bars represent the mean ± SD. Statistical significance was analyzed with unpaired Student's *t*-test (*p < 0.05, **p < 0.01, ***p < 0.001). **b** The mRNA expressions of down-regulated proteins, such as, ECM1, APOC1, S1OOA1O and ACSL4 of control and ischemia/reperfusion livers were verified using quantitative real-time analysis. The error bars represent the mean ± SD. Statistical significance was analyzed with unpaired Student's *t*-test (*p < 0.05, **p < 0.01, ***p < 0.001). **c** The mRNA expressions of five PPAR signaling pathway-related proteins Perlipin, PCK1, APOA5, SCD and ACS were detected. **d** Western blot analysis of AXL, PCK1, Perlipin, CRP, ECM1 and SCD expressions of control livers (C1, C2 and C3) and ischemia/reperfusion livers (S1, S2 and S3). **e** Representative western blot analysis of ECM1 and AXL protein levels in livers from mice subjected to sham treatment or ischaemia for 1 h followed by reperfusion for the indicated times

High-throughput techniques were very effective in elucidating the differential abundance of proteins in the hepatic I/R and control groups. To confirm the proteomic results and further extend our analysis of differentially expressed proteins, we analyzed both sets of samples using PRM-based validation proteomics, real-time quantitative PCR, and western blotting. The PRM data showed that the average number of individuals observed per group was consistent with the ratio of pooled samples (Table 2 and Additional file 4: Table S4). Next, we detected changes in the mRNA levels of the metabolic and inflammation-associated differentially expressed proteins ECM1, APOC1, S100A10, ACSL4, AXL, ARHGAP9, AIF1, ORM1, NUCB2 and HEPACAM (Fig. 5a, b). The PPAR signaling cascade was found to be perturbed in the human liver upon I/R treatment. Therefore, we focused on these proteins and verified their mRNA expression levels (Fig. 5c). RT-qPCR analysis showed that the mRNA levels of these proteins were consistent with our proteomic data (Fig. 5a–c). Then, we selected several representative upregulated proteins (AXL, PCK1, Perilipin and CRP) and downregulated proteins (ECM1 and SCD) and performed western blotting analysis, the results of which were also consistent with the proteomic data (Fig. 5d). Additionally, we analyzed ECM1 and AXL expression in liver lobes from mice subjected to liver I/R surgery. A gradual decrease in ECM1 expression and an increase in AXL expression from 1 to 24 h were observed after I/R surgery (Fig. 5e). Collectively, these data further verified the results of the proteomics experiment.

**Table 2:** Comparison between TMT and PRM results

Protein accession	Protein gene	S/C ratio(PRM)	S/C ratio(TMT)
P60908	S100A10	0.56	0.76
P02654	APOC1	0.57	0.76
P02763	ORM1	1.39	1.38
P80303	NUCB2	1.55	1.46

## Discussion

Tandem mass tag (TMT) proteomics has emerged as a new method of protein mass spectrometry and has been widely used for protein quantification and identification in the fields of cancer research [14, 15], neurobiology [16] and tissue analysis, such as biomarker research [17, 18]. Accumulating evidence indicates that reperfusion following hepatic ischemia gives rise to secondary injury accompanied by structural and functional damage. However, the molecular mechanisms underlying these phenotypes remain incompletely characterized. Only a limited number of proteomic studies of liver I/R tissues were reported more than a decade ago. Previous studies used proteomic approach to identify differences in protein expression between murine subjected to hepatic ischemia and sham treatment [19, 20]. In addition, only a few databases supported reliable quantification due to the limitations of mass spectrometry at that time [21]. Novel tools that allow us to take a high-throughput and quantitative approach to determining differentially expressed proteins are now available. Therefore, we decided to apply TMT-based quantitative high-throughput mass spectrometry to characterize the proteins that are differentially expressed in the human liver during ischemia/reperfusion. A total of 5,257 proteins were quantitatively identified. Among these, 142 proteins were significantly upregulated, while 103 proteins were significantly downregulated. The upregulated proteins were mainly involved in the oxidative, immune and inflammatory pathways, and the downregulated proteins were closely associated with metabolism. Upon hepatic I/R injury, major enzymes involved in lipid and steroid synthesis, cholesterol biosynthesis, energy metabolism and amino acid metabolism were downregulated, indicating the global impairment of metabolic activity in liver tissues, which might reflect an adaptive mechanism by which the hepatocyte reduces energy and nutrient dissipation under conditions of ischemia and reperfusion. Consistently, our studies confirmed previously reported changes in the oxidative pathway upon I/R injury [20, 22]. In addition, a recent study reported that a liver-selective MMP9 inhibitor eliminated ischemia/reperfusion injury and accelerated liverregeneration in rats [23]. Our study provides proteomic evidence to confirm that MMP9 expression is significantly increased in the human liver upon I/R.

However, though the great majority of proteins whose expression is altered in the liver upon I/R are enzymes involved in metabolic pathways, energy and lipid metabolism, the significant involvement of the immune and inflammatory pathways during the I/R response is particularly evident. For instance, allograft inflammatory cytokine-1 (AIF-1) was observed to be significantly elevated after hepatic ischemia/reperfusion, which is consistent with the results of a previous report in a rat model [19]. The expression of AIF-1, which modulates the immune response during macrophage and neutrophil activation, is upregulated by the cytokine IFN-γ [24]. AIF-1 has been reported to increase the production of IL-6, IL-10, and IL-12p40 compared with control cells. AIF-1 is overexpressed in liver allografts with acute rejection after transplantation in rats [25]. We hypothesized that AIF-1 aggravates hepatocyte injury in I/R via promoting inflammation. Importantly, however, the results of TMT-based quantitative proteomics analysis in our study demonstrated striking differences in other immunity- and inflammation-related proteins that have not been reported in the context of ischemia/reperfusion. Platelet factor 4 (PF4) is a small cytokine belonging to the CXC chemokine family that is also known as chemokine (C-X-C motif) ligand 4 (CXCL4). This chemokine is released from the alpha granules of activated platelets during platelet aggregation and promotes blood coagulation by moderating the effects of heparin-like molecules. Due to these roles, PF4 is predicted to play a role in inflammation [26]. C-reactive protein (CRP) was initially found to be secreted by pathogens since its level was elevated in a variety of illnesses, including cancer [27]. The later discovery of its hepatic synthesis (CRP is made in the liver.) demonstrated that CRP is a native protein. CRP is an acute-phase protein of hepatic origin that increases following IL-6 secretion by macrophages and T cells [28]. CRP is used mainly as a marker of inflammation in clinical diagnosis [27]. In the present study, we observed the increased expression of PF4 and CRP in the human liver upon I/R, suggesting that these proteins whose expression was enhanced aggravate hepatocyte injury during I/R through inducing inflammation.

AXL, a cell surface receptor tyrosine kinase, is characterized by an extracellular structure consisting of two fibronectin type 3-like repeats and two immunoglobulin-like repeats along with its intracellular tyrosine kinase domain. Signaling pathways activated downstream of AXL include the PI3K-AKT-mTOR, NF-kB and JAK/STAT pathways. AXL inhibits the innate immune response. The functions of activated AXL in normal tissues include the efficient clearance of apoptotic material and the inhibition of TLR-dependent inflammatory responses and natural killer cell activity [29]. ECM1 is a glycosylated protein secreted extracellularly that was found to contribute to migration and invasion in cholangiocarcinoma cells. Indeed, ECM1 has been shown to inhibit the activity of MMP9 [30]. MMP6 plays deleterious role in ischemia/reperfusion [31]. Therefore, elevating ECM1 or AXL levels could be a therapeutic strategy to ameliorate hepatic I/R injury. SASH1 is a scaffold protein involved in the TLR4 signaling pathway that may stimulate cytokine production and endothelial cell migration. The encoded protein has also been described as a potential tumor suppressor that may negatively regulate the proliferation, apoptosis, and invasion of cancer cells. Thus, the increased levels of these immunity-related proteins indicated their potential roles in I/R injury. This finding underscores the importance and possible link between inflammation and immunity in hepatic ischemia/reperfusion injury.

Peroxisome proliferator-activated receptors (PPARs) are a family of nuclear receptors that comprises ligand-modulated transcription factors with broad tissue distributions and a wide array of target genes and functions [32]. PPARs are naturally activated by lipid-derived substrates to increase the proliferation of peroxisomes [33]. The PPAR subfamily is composed of three subtypes: PPARα, PPARδ/β, and PPARγ. PPARα is expressed mainly in tissues with high fatty acid metabolism, including the liver, kidney, and white and brown adipose tissue. PPARδ/β, which is ubiquitously and abundantly expressed in a broad range of tissues, regulates fatty acid oxidation. PPARγ is expressed in various cell types, including brain cells and bone marrow-derived immune cells. PPARγ is a ligand-activated nuclear receptor that regulates glucose and lipid metabolism, endothelial function and inflammation [34]. Here, by an initial manual query, the expression levels of the following six proteins associated with the PPAR signaling cascade were found to be significantly altered after hepatic ischemia/reperfusion: SCD, Perilipin, FABP, ACS, APOA5 and PEPCK (Additional file 7: Figure S3). Hepatic I/R appears to promote the expression of fatty acid binding Protein 3 (FABP3), SCD, Perilipin-1, PEPCK and APOA5 and inhibit the expression of ACS, indicating that the PPAR signaling cascade is perturbed in the human liver upon I/R treatment. Taken together, our observations support the hypothesis that the liver undertakes protective strategies after ischemia/reperfusion by upregulating immunity- and inflammation-associated functions and downregulating metabolism.

In this study, these differentially expressed proteins were observed within 30 min of initiation of hepatic I/R injury, which is consistent with previous literatures. He W et al. reported that expression of TXNIP is elevated in rat livers exposed to 30 min of warm ischemia [35]. Nicoud IB et al. harvested livers tissues during ischemia or after early reperfusion time points from mice subjected to 30 min of 70% hepatic ischemia. The result showed that sham-operated liver tissue and ischemic liver tissue harvested before reperfusion do not express MMP9; however, reperfusion for 15 or 30 min resulted in observable MMP9 in the liver [36]. Olthof PB et al. reported that Foxp3 is more strongly upregulated in the 30-min ischemia group compared to the 60 min ischemia group. Foxp3 is the master regulator of regulatory T cells, which are induced by cytokines such as TGF-β, inhibit the development of pro-inflammatory effector T cells. Also, regulatory T cells are protective in hepatic I/R, which may play a role in reducing inflammatory response. Furthermore, they made the point that mouse models using ≤ 30 min of ischemia best reflect the clinical liver I/R injury profile in terms of liver function dynamics and type of immune response [37].

The current study has some limitations. First, for obvious reasons, one limit of this study was the inability to control the ischemia and reperfusion times during hepatectomy. Second, due to interindividual variability in the cellular response to I/R injury, further studies will be devoted to evaluating the role of these specific proteins in a larger number of clinical cases. Finally, the concentrations of the relatively abundant proteins identified here are only one factor in cellular reactions to stimuli. Posttranslational modifications are important components of cellular responses that will need to be addressed in future studies.

In summary, our findings revealed multiple protein changes potentially involved in hepatic I/R injury in human samples. We found that the liver upregulates immunity- and inflammation-associated functions and downregulates metabolism to undertake protective strategies upon ischemia and reperfusion stimuli. In particular, we identified a series of proteins that had not previously been reported, PF4, CRP, AXL, ECM1, SASH1, APOC1, ACSL4, ORM1 and NUCB2, as well as members of the PPAR signaling pathway. We anticipate that further studies of the proteins identified herein will expand our understanding of the mechanisms of hepatic I/R injury, thus assisting in the eventual development of new therapeutic approaches against hepatic I/R injury.

## Supplementary Information

**Additional file 1: Table S1.** The list of  significantly regulated proteins.

**Additional file 2: Table S2. **Real-time PCR primers sequences.

**Additional file 3: Table S3.** GO terms level classify.

**Additional file 4: Table S4. **The peptides of the inclusion list in PRM assay.

**Additional file 5: Figure S1.** Functional enrichment analysis based on protein domains of differentially expressed proteins upon hepatic I/R treatmemt.

**Additional file 6: Figure S2.** The clustering analysis heat map based on GO enrichment includes three categories: **a** Biological Process **b** Cellular Component and **c** Molecular Function **d** Cluster analysis heat map based on KEGG pathway enrichment.

**Additional file 7: Figure S3.** Significantly enriched differentially expressed proteins were visualized in the PPAR signaling pathway.
