# Supplementary material for: Global proteome profiling of human livers upon ischemia/reperfusion treatment
Source: Clin Proteomics. 2021 Jan 6;18:3. doi: 10.1186/s12014-020-09310-w (PMC7788958; doi:10.1186/s12014-020-09310-w)
Supplement: Supplementary file 2 — Additional file 2: Table S2. Real-time PCR primers sequences. [file 12014_2020_9310_MOESM2_ESM.doc]

**Supplementary table 2. Real-time PCR primers sequences**

| ECM1 | **Forward** -CTGGCTAGGAGAAAGGGTGG  **Reverse** -TCTTTTTGGAGGGGGACAGC |
| --- | --- |
| APOC1 | **Forward** -GGGAAAGGGACTAAGGTGGT  **Reverse** -GGGGCACTCTGAATCCTTG |
| S100A10 | **Forward** -ACAAGAACGCTCTGTCTGGG  **Reverse** -TGCGCCTTCCTTAGTACGTG |
| ACSL4 | **Forward** -GGGGATGGGGGAGTTGTTTT  **Reverse** -TTTACCCACACCACGGAAGG |
| AXL | **Forward** -CAGTGCCAAATCCGGGGAG  **Reverse** -AGGGACTTTCTTCAGCCTGC |
| ARHGAP9 | **Forward** -ACCTTGTGTCTCCCTGTTTCTG  **Reverse** -AGTTGGTCCTGGGTAGTGGT |
| AIF1 | **Forward** -CTCCAGCTTGGAGGAAAAGC  **Reverse** -TGGAGGGCAGATCCTCATCA |
| ORM1 | **Forward** -GACAAGCCAGAGACGACCAA  **Reverse** -ACTGTCCAATCCCTTGCGTT |
| NUCB2 | **Forward** -CCCAATCCCTTTTGCCCAGA  **Reverse** -GTACGCCTCGTATTCGGTCC |
| HEPACAM | **Forward** -GCATCTTCCTCCTTGTGACCT  **Reverse** -GAGGGTGTCTGCTTCTGGTT |
| SCD | **Forward** -CTTGCGATATGCTGTGGTGC  **Reverse** -CCGGGGGCTAATGTTCTTGT |
| Perilipin | **Forward**-GAGTGAGTGTTGGGGTCCTG  **Reverse** -TCAGGGAGGTCTCCATCCAG |
| ACS | **Forward**-GAGGCCCCGCCTCTAGTTC  **Reverse** -AAGCCCCATCACGTCAAGTT |
| PCK1 | **Forward**-CACACTTGAAGAGGGGGTGC  **Reverse** -GGGATACAGAAGGCGCTCAA |
| APOA5 | **Forward**-CCTCCCTCCACCTGTCTTCT  **Reverse** -AAGGCTGTCTTTCAGGGTCG |
| GAPDH | **Forward**-ACCACAGTCCATGCCATCAC  **Reverse** -TCCACCACCCTGTTGCTGTA |
