# Supplementary material for: Global proteome profiling of human livers upon ischemia/reperfusion treatment
Source: Clin Proteomics. 2021 Jan 6;18:3. doi: 10.1186/s12014-020-09310-w (PMC7788958; doi:10.1186/s12014-020-09310-w)
Supplement: Supplementary file 4 — Additional file 4: Table S4. The peptides of the inclusion list in PRM assay. [file 12014_2020_9310_MOESM4_ESM.docx]

**Supplementary Table 4. The peptides of the inclusion list in PRM assay**

| Peptide | Retention Time | Protein Accession | Protein Gene | C_mix Area | S_mix Area | C_mix Normalized Area | S_mix Normalized Area |
| --- | --- | --- | --- | --- | --- | --- | --- |
| EFPGFLENQK | 32.36 | P60903 | S100A10 | 4847700.00 | 2701600.00 | 1.28 | 0.72 |
| EFGNTLEDK | 14.00 | P02654 | APOC1 | 23480000.00 | 9349800.00 | 1.43 | 0.57 |
| EWFSETFQK | 32.37 | P02654 | APOC1 | 7072500.00 | 5567900.00 | 1.12 | 0.88 |
| EQLGEFYEALDCLR | 47.36 | P02763 | ORM1 | 10412000.00 | 9922600.00 | 1.02 | 0.98 |
| SDVVYTDWK | 23.52 | P02763 | ORM1 | 16000000.00 | 33244000.00 | 0.65 | 1.35 |
| VQNIHPVESAK | 7.46 | P80303 | NUCB2 | 6068000.00 | 9377500.00 | 0.79 | 1.21 |
