# Supplementary figures and images for: Global proteome profiling of human livers upon ischemia/reperfusion treatment
Source: Clin Proteomics. 2021 Jan 6;18:3. doi: 10.1186/s12014-020-09310-w (PMC7788958; doi:10.1186/s12014-020-09310-w)

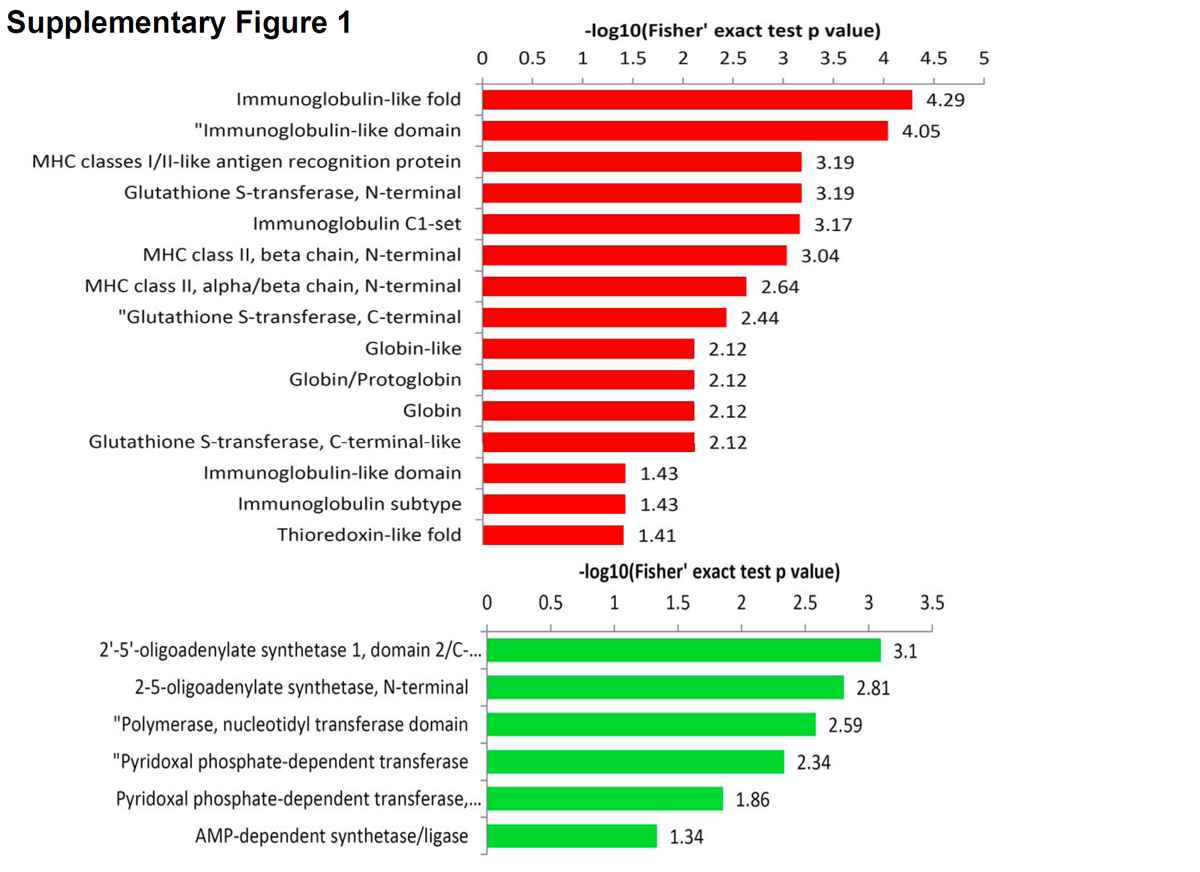

Supplement: Supplementary file 5 — Additional file 5: Figure S1. Functional enrichment analysis based on protein domains of differentially expressed proteins upon hepatic I/R treatmemt. [file 12014_2020_9310_MOESM5_ESM.tif]

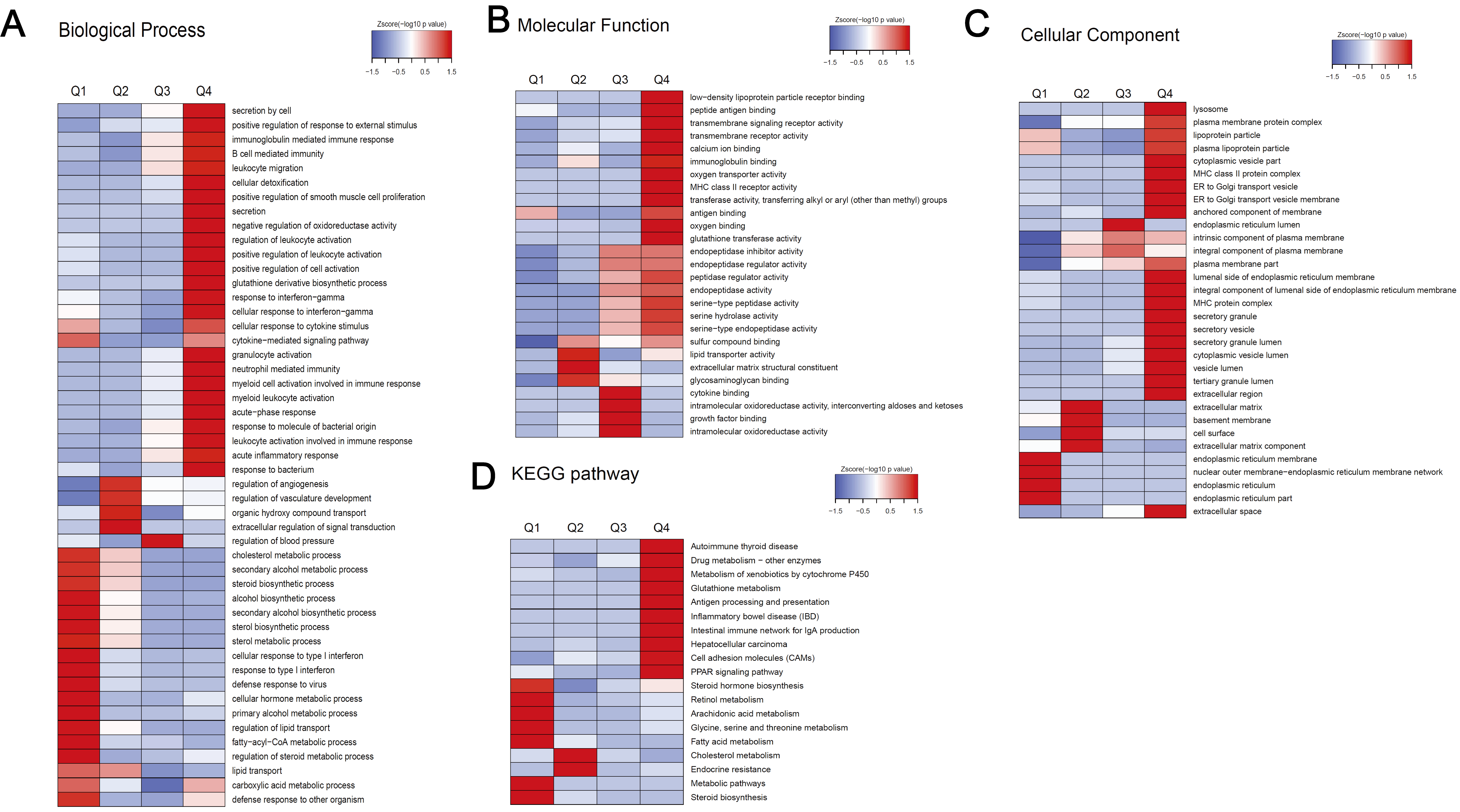

Supplement: Supplementary file 6 — Additional file 6: Figure S2. The clustering analysis heat map based on GO enrichment includes three categories: a Biological Process b Cellular Component and c Molecular Function d Cluster analysis heat map based on KEGG pathway enrichment. [file 12014_2020_9310_MOESM6_ESM.tif]

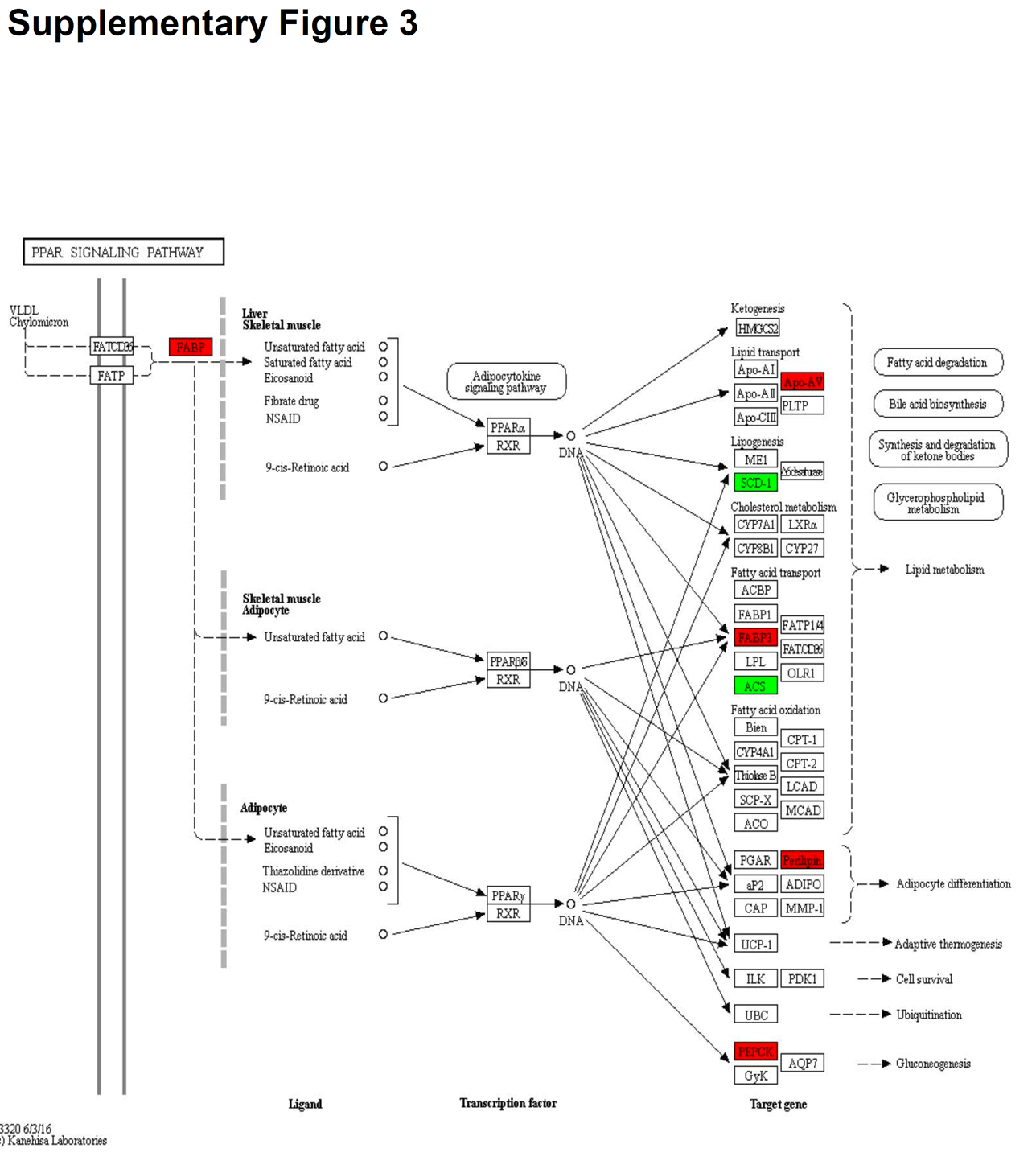

Supplement: Supplementary file 7 — Additional file 7: Figure S3. Significantly enriched differentially expressed proteins were visualized in the PPAR signaling pathway. [file 12014_2020_9310_MOESM7_ESM.tif]
